# Histological, immunohistochemical assessment and DNA fingerprint species identification of some meat products in Egypt

**DOI:** 10.1038/s41598-025-97633-9

**Published:** 2025-04-29

**Authors:** Heba F. Kamaly, Abeer M. Hassan, Zainab MA Youssef, Fatma El-Zahraa Ahmed Mustafa

**Affiliations:** 1 Department of Forensic Medicine and Toxicology, Faculty of Veterinary Medicine, Assiut University, 71526 Assiut, Egypt; 2 Department of Food Hygiene, Safety and Technology, Faculty of Veterinary Medicine, Assiut University, 71526 Assiut, Egypt; 3 Infectious Diseases, Department of Animal Medicine, Faculty of Veterinary Medicine, Assiut University, 71526 Assiut, Egypt; 4 Department of Cell and Tissues, Faculty of Veterinary Medicine, Assiut University, Assiut, 71526 Egypt

**Keywords:** Adulteration, Histological analysis, Bcl2 expression, DNA fingerprint, Meat products, Cell death, Cell biology, Anatomy

## Abstract

A total of sixty commercial beef products, represented by minced meat, sausage, kofta, and burger, with fifteen samples per product, were collected randomly from different markets in Assiut city, Egypt. Samples were examined histologically, immunohistochemically and molecularly to investigate tissue composition and species substitution. Polymerase Chain Reaction (PCR) was applied to confirm the beef origin of different marketed beef products and determine if there are any adulteration and/or contamination with rodents and canine species. The histological investigation finds significant differences in skeletal muscle content, with the highest proportion in minced meat, whereas the lowest detected in kofta. Several animal tissues were detected, including adipose tissue, collagen, cartilage, and bone, where kofta showed the highest levels. We also detected plant tissues, predominantly found in burger samples. Expression Bcl2 indicated the maximum intensity in sausage, while burger showed the lowest expression. PCR results revealed that 89.13% were pure beef products, 10.87% were with rat meat contamination, and 100% of examined samples were negative for canine species. These results highlight the efficacy of histology, Bcl2 immunohistochemistry and PCR in assessing meat quality and distinguishing adulteration.

**Supplementary Information:**

The online version contains supplementary material available at 10.1038/s41598-025-97633-9.

## Introduction

Meat is favored worldwide for its nutritive value, which is rich in protein, vitamins, minerals, and fat needed for the human body^1,2^. In Egypt, the absence of a monitoring program, low awareness of the consumer, increased population, demands for animal protein, and low economic conditions encouraged the food adulteration or/and contamination, especially of minced products^3–5^.

The high price of meat forced Egyptians to replace it with meat products regardless of their ingredients and quality. According to the European Union labeling regulations, any additive ingredients in the meat products must be mentioned in their label to ensure the assent and awareness of the consumer, where the absence of these ingredients in the label is considered fraud and adulteration^6^.

Adulteration in the meat industry is considered one of the most important issues related to human health and safety^7^. There are different purposes and pathways of adulteration, such as the following:

Intentional adulteration, which is done through foreign ingredient additions that are not included in the product^’^s label, aims to increase the product’s weight or improve its appearance. Fraud additives are either of animal origin, which may cause disease transmission to humans, or plant origin, such as soybean, which may act as an allergen and lead to a decrease in the product’s quality^8,9^.

Unintentional contamination of meat and its products with unacceptable meat species as rodents indicates the bad hygienic conditions in these factories and may be responsible for transmission of infectious diseases to humans^10–12^.

Since food fraud is a relatively common practice around the world, there are different accurate and rapid methods to detect the adulteration of meat and its products and the extent of its acceptability. Chromatography, mass spectrophotometry, and serology analytical methods can only detect proteins and metabolites of meat without the determination of species or tissue substitution^13^.

Histological analysis is a newly used method that appears as an effective method to describe tissue composition and detect various unauthorized tissues qualitatively and quantitatively^14–16^. According to available data, no available literature study uses immunohistochemistry to assess meat quality in meat products. So, we insert immunohistochemistry using Bcl2 expression as a new approach to meat quality assessment by detecting apoptotic cells.

Apoptosis, or programmed cell death, is crucial for the cells’ selective elimination, a method that supports cellular equilibrium in tissues^17^. There were two main known pathways for apoptosis, intrinsic and extrinsic. In addition, once apoptosis is initiated, it cannot be reversed^17,18^. Regulation of both pathways of apoptosis was regulated and controlled by various molecules, and one of the key players in this process was the Bcl- 2 family, which is considered as proteins important in the regulation of cell apoptosis and survival^19^. Moreover, mitochondrial apoptotic signaling in skeletal muscle is regulated with proteins of the BCL2 family^20^.

Molecular analysis is considered an old, effective method for adulteration and/or contamination detection because the PCR technique is a potential molecular technique that is vital for meat species identification in the food of animal origin^3,21,22^. Genetic species identification with PCR is considered the most rapid, reliable, and sensitive monitoring test to detect the deliberate adulteration and/or unintended contamination of meat with any traces of tissues even if cooked for religious and health implications^23^, where in Islam, dead animals or pork tissues are strictly banned to ingest as well as the beef for the Hindus^24^.

There are numerous previous studies that illustrated the prevalence of adulteration in meat products in Egypt and the role of the PCR technique for species detection, such as the study performed in various regions in Upper Egypt, which showed the mislabeling and adulteration of different commercial beef meat products with chicken, donkey, and human tissues^25^. In Kalubia governorate, Egypt, adulteration was investigated in beef kofta and beef sausage with canine meat^26^. In Cairo governorate, Egypt, a previous study showed that 16.4% of commercial beef products samples were negative for bovine species, and 9.2% of the analyzed products were adulterated with rodents^27^. Illegal additions of equine and canine meat in beef-based meat products collected from the Cairo and Menoufiya governments in Egypt were detected^28^. Also, a previous investigation described chicken, camels, donkeys, and pig species in beef-based meat products collected from El-Fayoum, Egypt^29^.

The current study aimed to evaluate the quality and adulteration of some consumed beef products, including minced meat, burger, sausage and kofta using different techniques such as histological analysis and Bcl2 expression immunohistochemistry, as well as species DNA-based identification with PCR molecular assay.

## Materials and methods

### Materials

#### Ethical approval

We obtained meat products from various markets in Assiut City, Egypt. We carried out all procedures in compliance with the applicable policies and guidelines. The Assiut University Faculty of Veterinary Medicine ethical committee approved this study (No. 06/2024/0279).

#### Samples collection

A total of 60 different beef product samples, including 15 samples from each product of burger, minced meat, kofta, and sausage, were randomly gathered from local and high-end markets in various regions of Assiut City, Egypt. Meat product samples were stored at -20°C for the analysis. All samples were fixed in formalin 10% for histological and immunohistochemical examination, and forty-six samples were analyzed with PCR to confirm the beef origin of meat products and to detect if there are any species adulteration or contamination by using positive control of bovine, rodents, and canines.

#### Chemicals

- ABT Genomic DNA Mini Extraction Kit (spin column with Cat. No. EX01), ABT 2X Red Mix (Cat. No. AMP 01), PCR grade nuclease free-water (Cat. No. EL03), ethidium bromide, 10x Tris borate EDTA (TBE) electrophoresis buffer, DNA gel 6x blue loading dye (Cat. No. EL04),100 bp ladder were obtained from Applied Biotechnology, Egypt.
- Primers for bovine, rodents, and canines were obtained from Integrated DNA Technologies, Belgium.
- High-Resolution Grade agarose, obtained from (Industrias Roko, S.A.).

## Methods

### Histological examination

After fixation, we immersed the fixed samples in ethyl alcohol for dehydration, then methyl benzoate used for sample clearing. the cleared samples embedded in paraffin wax and cut at a thickness of 3–5 μm. Harris haematoxylin and eosin histological stains (H&E) were used to stain the Sect^30^.

### Immunohistochemistry

Immunohistochemistry was performed on 3–5 μm-thick sections of sausage, burger, kofta, and minced meat. We washed the sections for 5 min with PBS (pH 7.4) after dewaxing and rehydrating them. Also, deactivation of endogenous peroxidase was performed using H2O2 (3%) for 20 min, then the section was washed with water for 10 min, and in a 95–98 °C water bath, samples were placed in a sodium citrate buffer solution (pH 6.0). At room temperature, sections of sausage, burger, kofta, and minced meat were washed in PBS (pH 7.4) and stained with: Bcl- 2 (B-cell lymphoma), Antibody (1:200; Catalog No. A16776; ABclonal)^31,32^,. OLYMPUS DP72 camera and OLYMPUS BX51 microscope were used for figures taken.

### Morphometric measurements

Using Image-J software, we take various morphometric measurements:

- Percentage (%) of SKM, animal tissues, and plant tissues present in samples of sausage, burger, kofta, and minced meat:
- Percentage intensity of BCL2 in sausage, burger, kofta, and minced meat (%) as the following: First, 8-bit image were obtained from the image column. Then, from the Analyze column, choose area fraction and area. Finally, from the image column, choose adjust, then threshold^33^.

### Statistical analysis

The data were statistically analyzed. We statistically analyzed the data from the histological and immunohistochemical sections using SPSS (26.0) one-way ANOVA. To compare variables, Duncan’s multiple range test was used^34^.

## Polymerase chain reaction

### DNA extraction from meat products

For each collected meat product sample, 500 mg were homogenized in 2 ml saline solution by D-Series Benchtop Homogenizer then DNA was extracted organically according to ABT Genomic DNA Mini Extraction Kit (spin column) instructions.

### Primer sequences of different tested species

Identify animal species in different meat products according to Table 1 which showed the primers for bovine, rodents and canines.

**Table 1:** Primers of bovine, rodents, and canines with the sequences and size.

Animal species	Target gene	Sequence (5′ → 3′)	Size bp	Ref.
**Bovine**	F R	5´GCC ATA TAC TCT CCT TGG TGA CA3´ 5´GTA GGC TTG GGA ATA GTA CGA3´	271	^35^
**Rodents**	F R	5´AAT CCA ACT TAT ATG TGA AAA TTC ATT GT 3´ 5´ TGG GTC TTA GCT ATC GTC GAT CAT 3´	96	^36^
**Dog**	F R	5′ GGA GTA TGC TTG ATT CTA CAG ′3 5′ AGA AGT GGA ATG AAT GCC ′3	808	^37^

### PCR amplification

The amplification was performed on PCR thermocycler (Cole-Parmer, United States) where a 20µl reaction mixture was prepared using 10µl of ABT 2X Red Mix, 0.5µl of each primer, 4µl PCR grade nuclease-free water and finally 5µl of target DNA of the sample. The amplification cycling conditions for different species according to Table 2 are the following

**Table 2:** The initial denaturation, amplification and final extension conditions of bovine, rodents and canine primers.

Species	Initial denaturation	Amplification (40 cycles)			Final extension	Ref.
		Secondary denaturation	annealing	Extension		
Bovine Rodents	95 ^o^C 2 min.	95 °C 20 s.	56 °C 30 s.	72 °C 45 s.	72 °C 10 min.	^27^
Canine	94^o^C 4 min.	94 ^o^C 60 s.	58 ^o^C 60 s.	72 ^o^C 60 s.	72 ^o^C 10 min.	^38^

### Electrophoresis and visualization of PCR amplified products 

The amplified products were visualized after the electrophoresis step, which was performed on agarose gel (2%), High-Resolution Grade agarose with ethidium bromide (1.5-2 μl of 10 mg/ml) in 10x Tris borate EDTA (TBE) electrophoresis buffer.

Five μl of PCR products were mixed with 3 μl of DNA gel 6x blue loading dye were injected in agarose gel, which was previously stained with ethidium bromide as well as, the 100bp ladder was used to determine the target fragment sizes by gel electrophoresis at 90 V and 155 mA for 60 or 70 min. Finally, gels were visualized and photographed on Gel UV transilluminator (Syngene, United Kingdom).

## Results

**Fig. 1:**
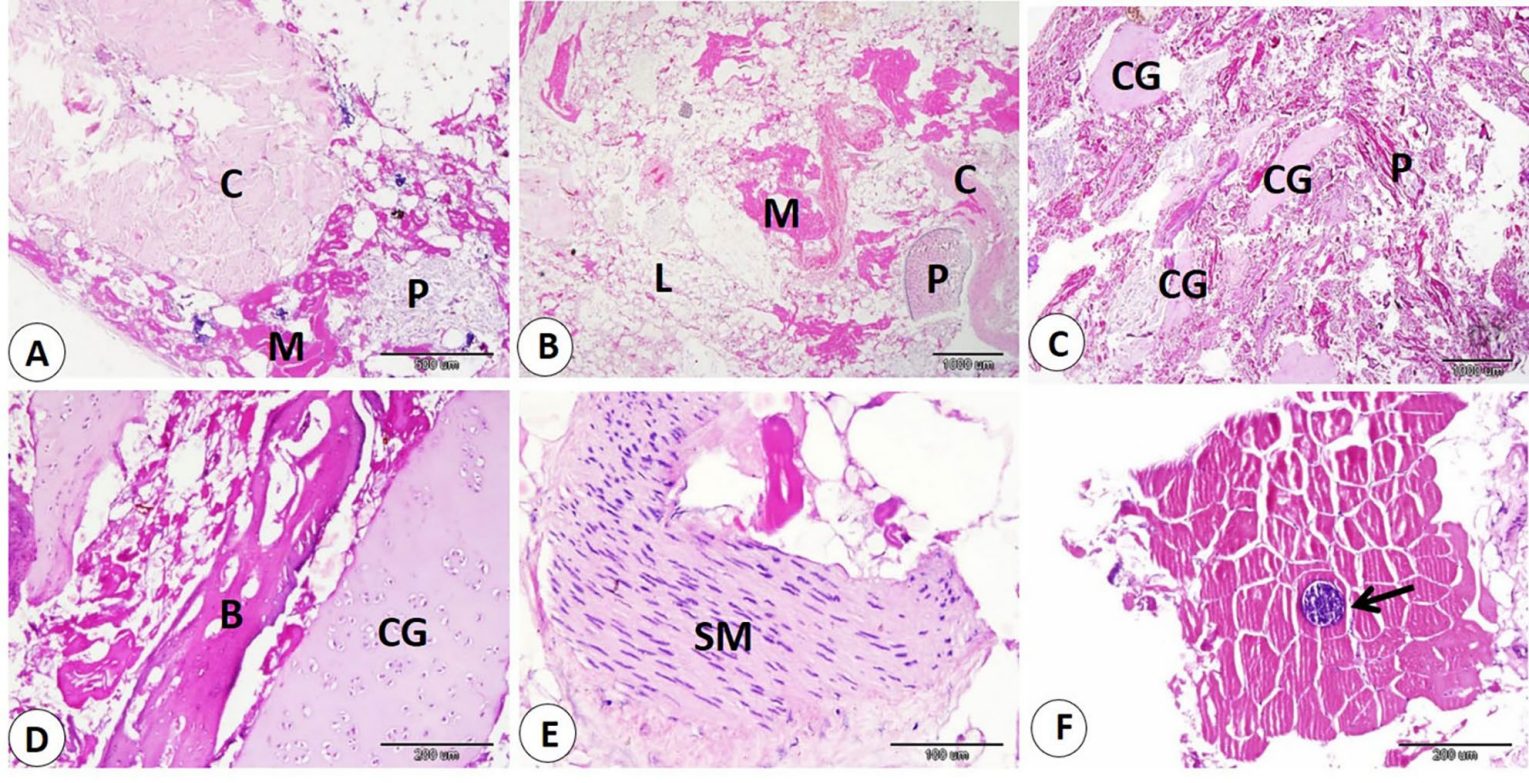
Samples of sausage showed adulteration with animal tissues. **A**: collagen (C), skeletal muscle (M), and plant tissue (P). H and E stain. **B**: lipid (L), skeletal muscle (M), collagen (C), and plant tissue (P). **C**: cartilage (CG), and plant tissue (P). D: cartilage (CG), and bone (B). **E**: smooth muscle (SM). **F**: sarcocyst (black arrow).

Histological examination of commercial sausage, burger, kofta, and minced meat detected adulteration with varying percentages of animal and plant tissues. Also, sarcocyst can observed in the skeletal muscle fibers. Our observation indicated significant differences in skeletal muscle content in examined products. The samples of minced meat showed the highest percentage of skeletal muscle which significantly increase than that of other products. On the other hand, the samples of kofta significantly decrease in percentage of skeletal muscle content than of the other products samples (fig. 1A-B-F, 3A-B,5A,7A-C) and (Table 3).

**Fig. 2:**
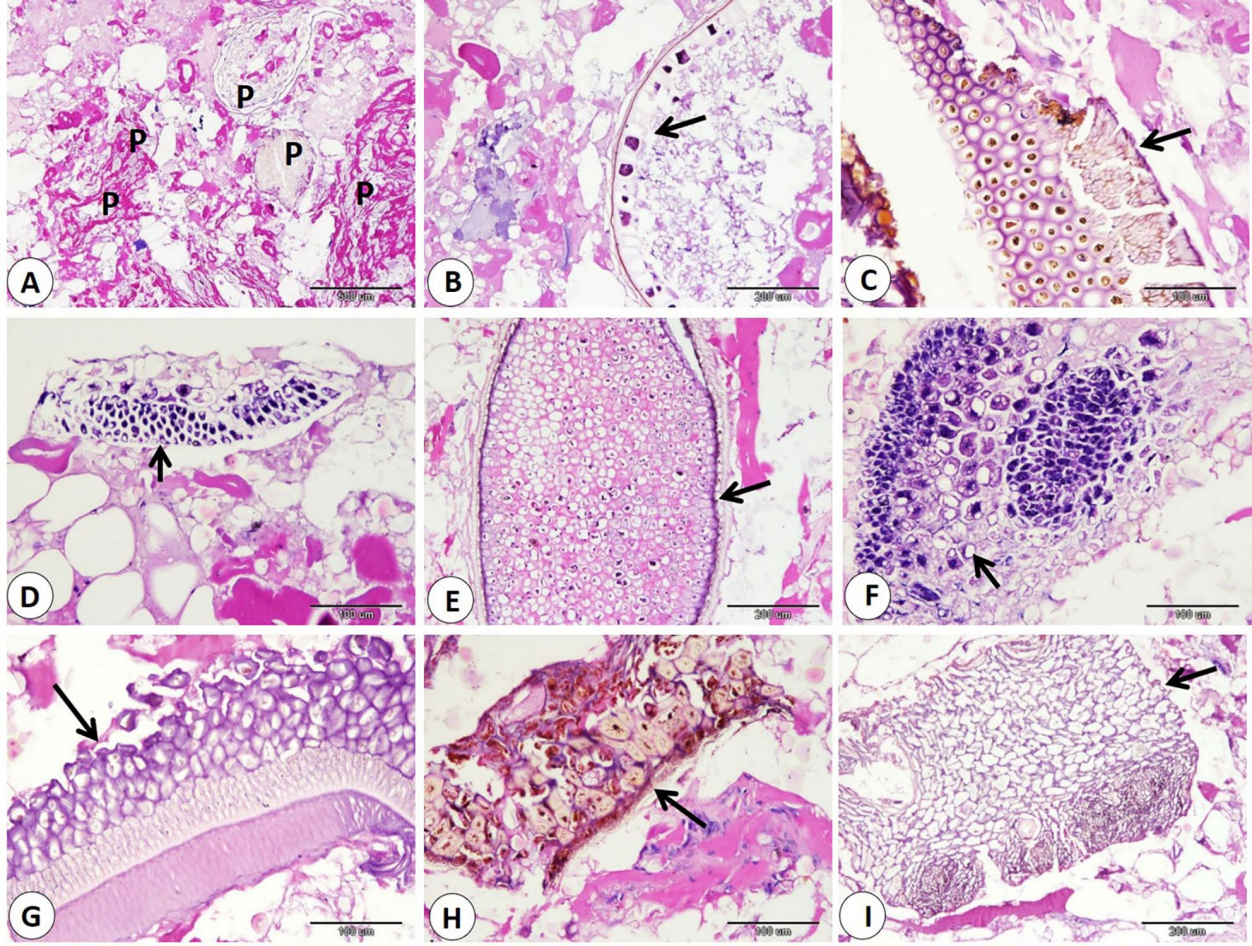
Samples of burger showed adulteration with animal tissues. **A**: lipid (L), collagen (C), and skeletal muscle (M). **B**: collagen (C), and skeletal muscle (M). **C**: bone (B), and plant tissue (P). **D**: smooth muscle (SM). H and E stain.

**Fig. 3:**
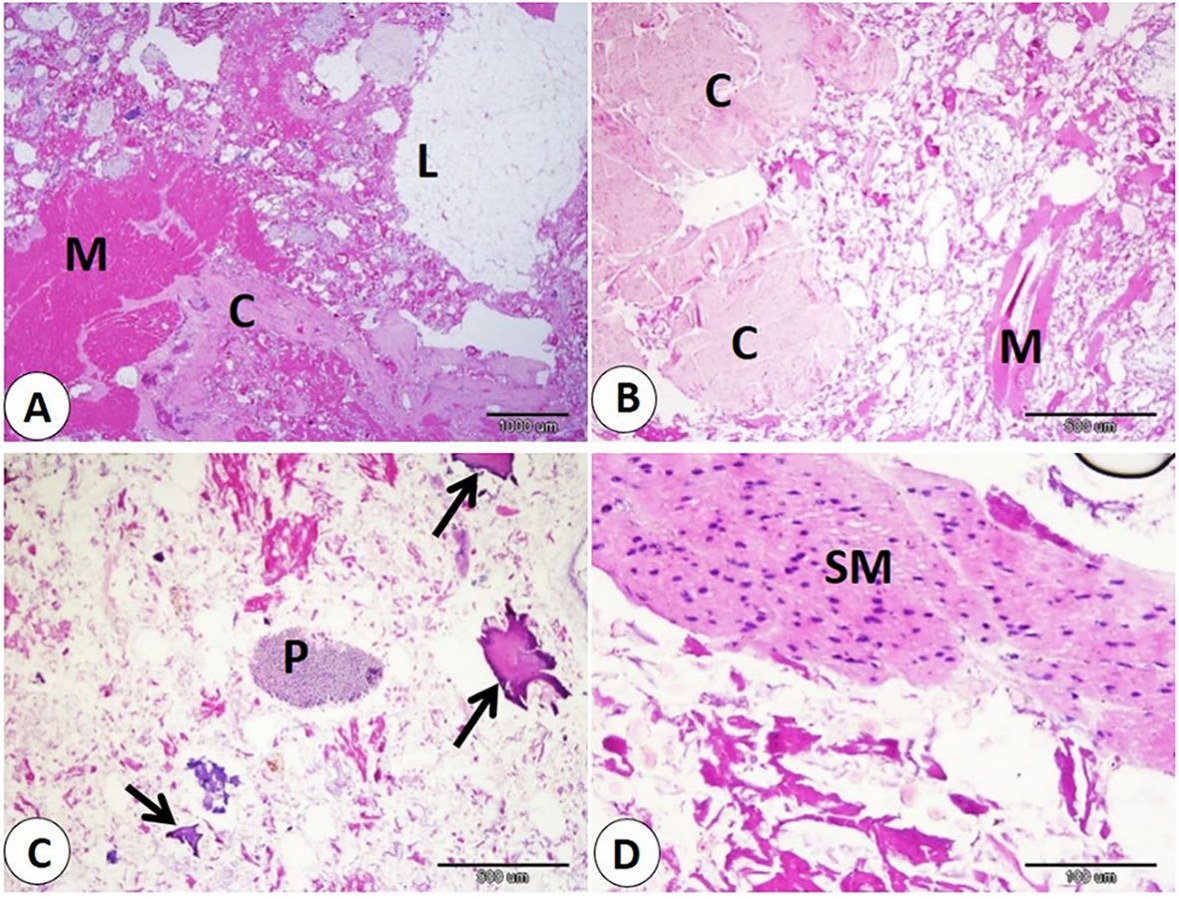
Samples of burger showed adulteration with animal tissues. **A**: lipid (L), collagen (C), and skeletal muscle (M). **B**: collagen (C), and skeletal muscle (M). **C**: bone (B), and plant tissue (P). **D**: smooth muscle (SM). H and E stain.

**Fig. 4:**
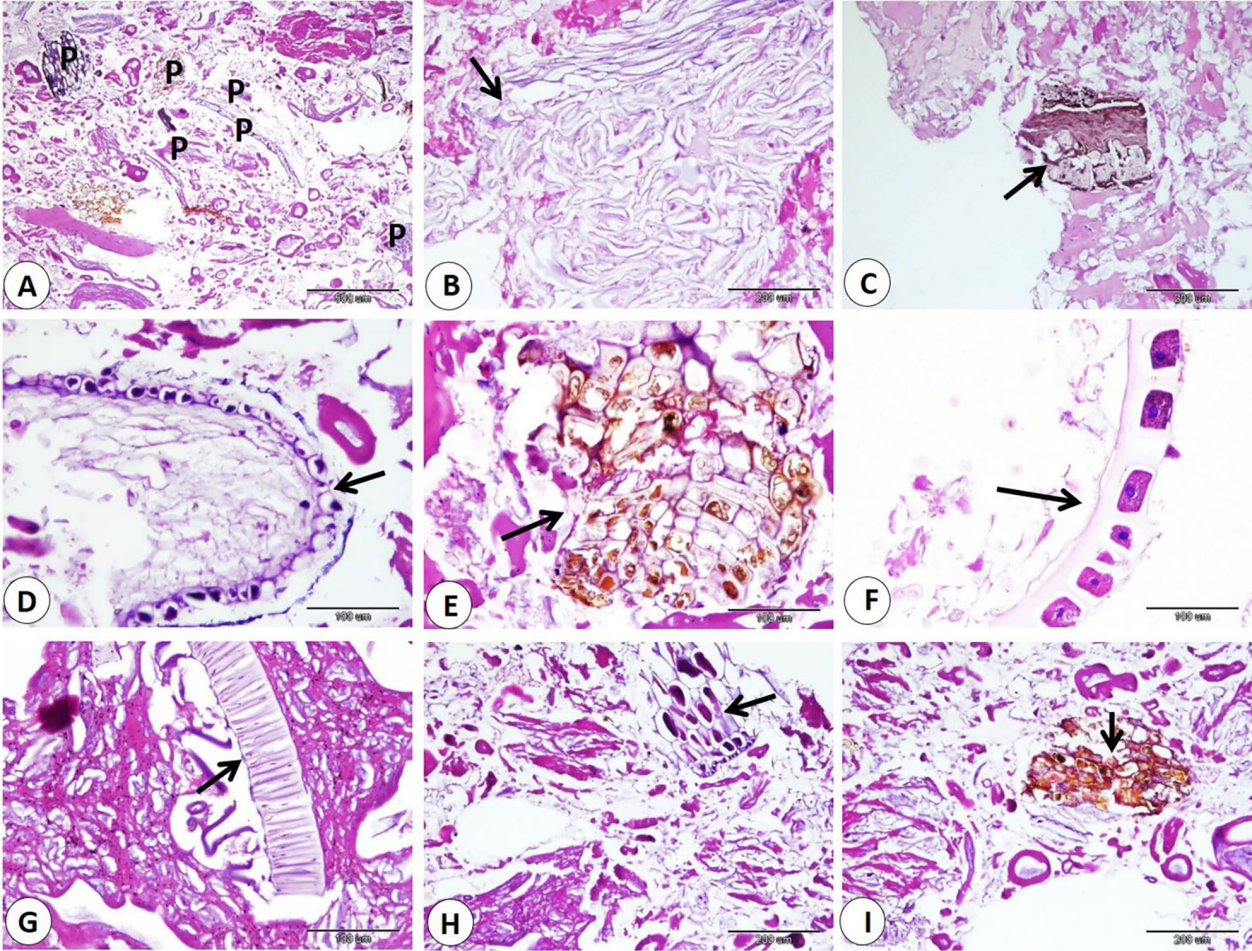
Samples of burger showed adulteration with different parts of plant tissues (arrows). H&E stain.

**Fig. 5:**
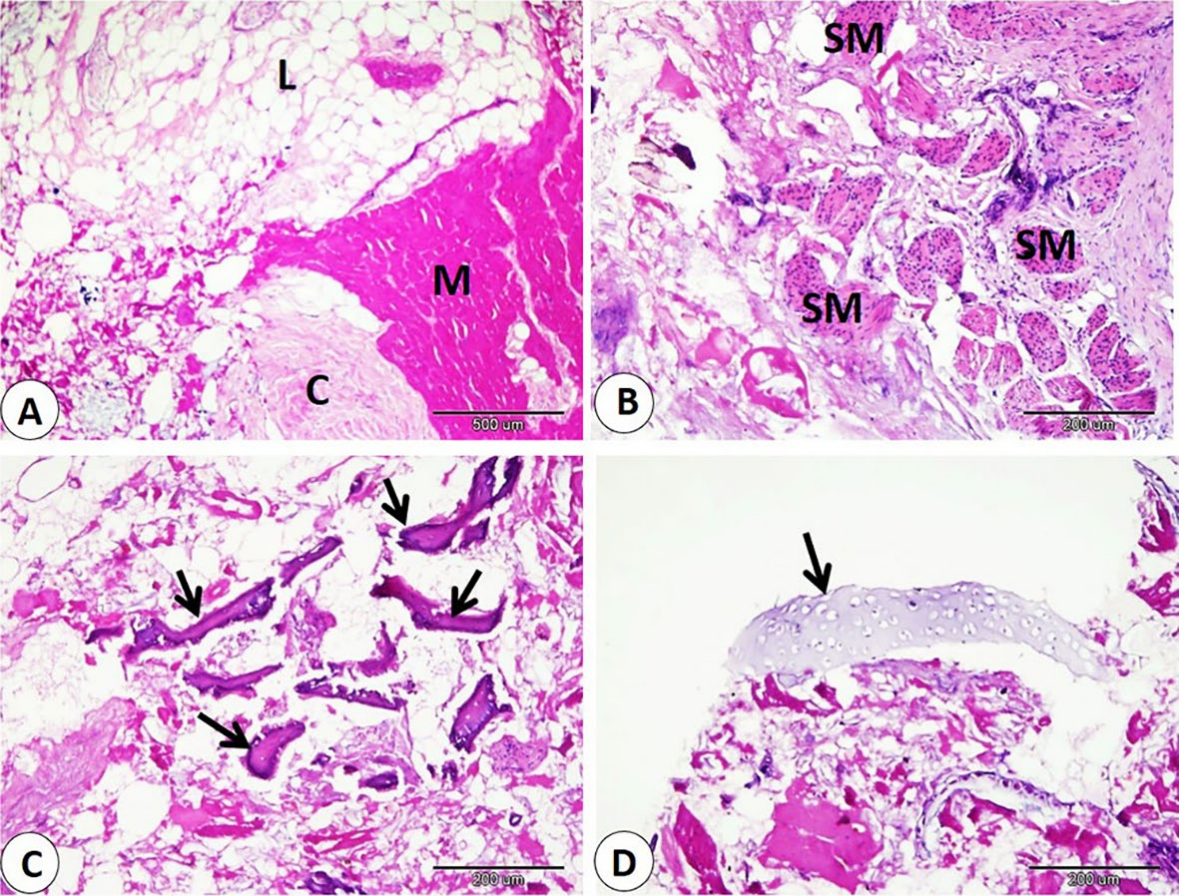
Samples of kofta showed adulteration with animal tissues. **A**: lipid (L), collagen (C), and skeletal muscle (M). **B**: smooth muscle (SM). **C**: bone (arrows). **D**: cartilage (arrow). H and E stain.

**Fig. 6:**
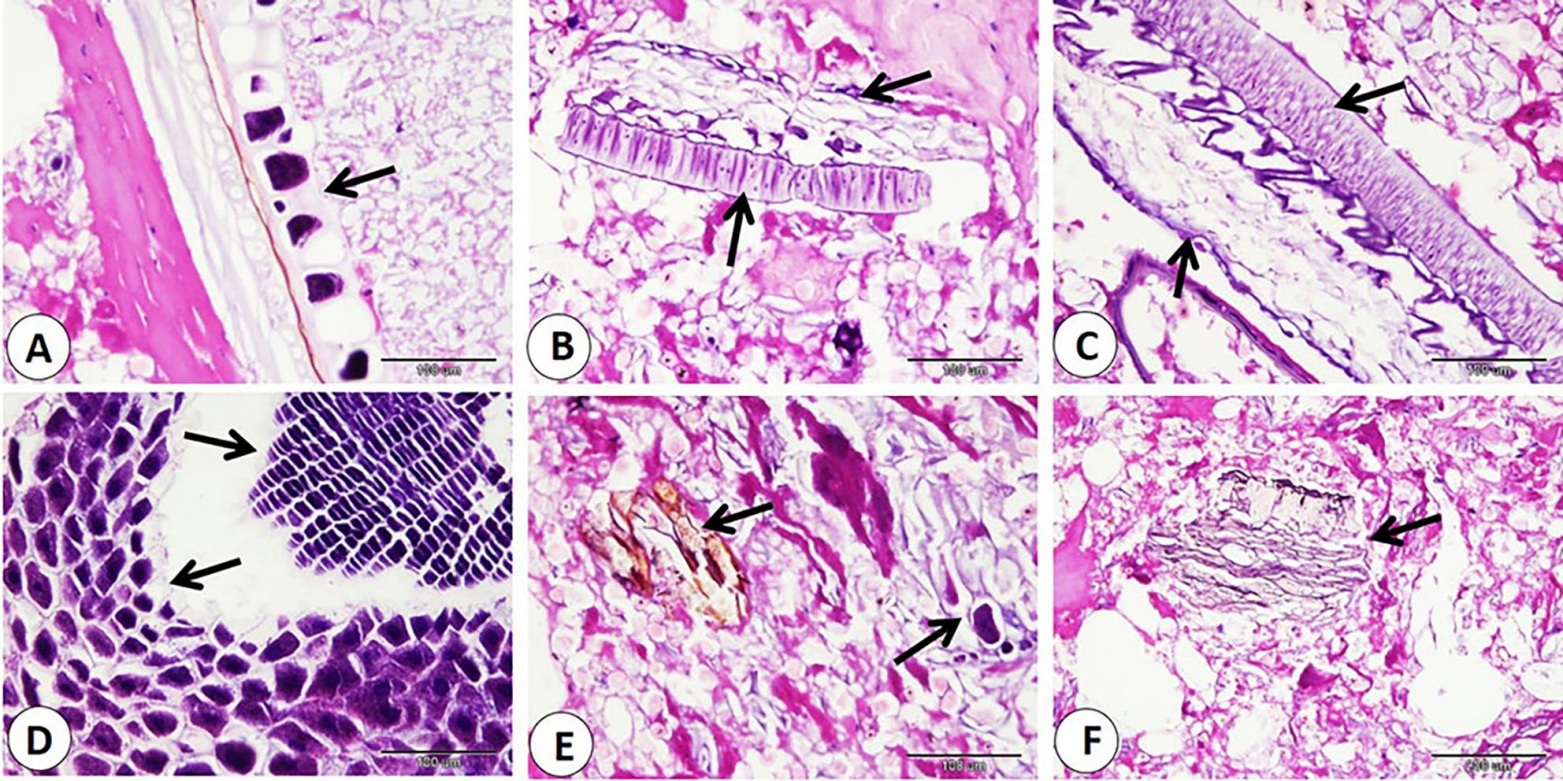
Samples of kofta showed adulteration with different parts of plant tissues (arrows). H&E stain.

**Fig. 7:**
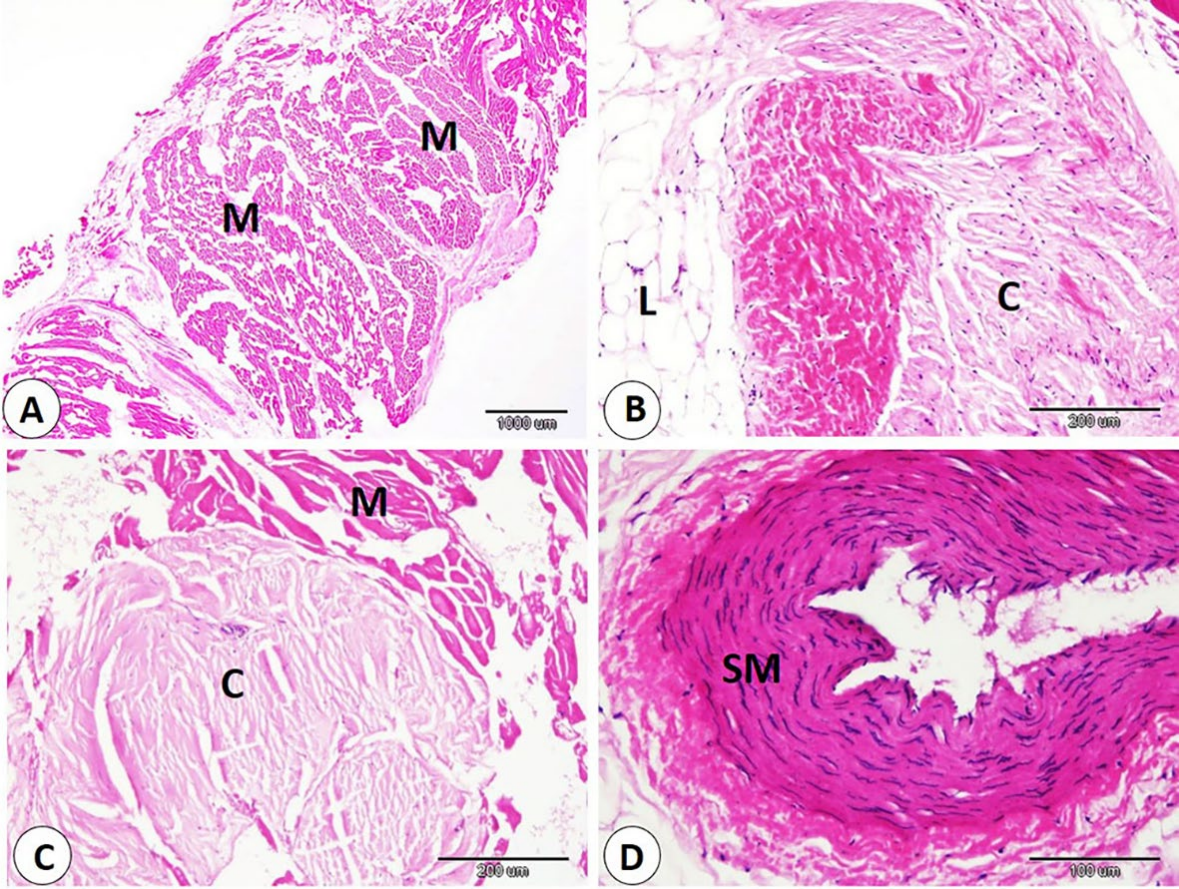
Samples of minced meat showed adulteration with animal tissues. **A**: skeletal muscle (M). **B**: lipid (L), and collagen (C). **C**: skeletal muscle (M), and collagen (C). **D**: smooth muscle (SM). H&E stain.

**Fig. 8:**
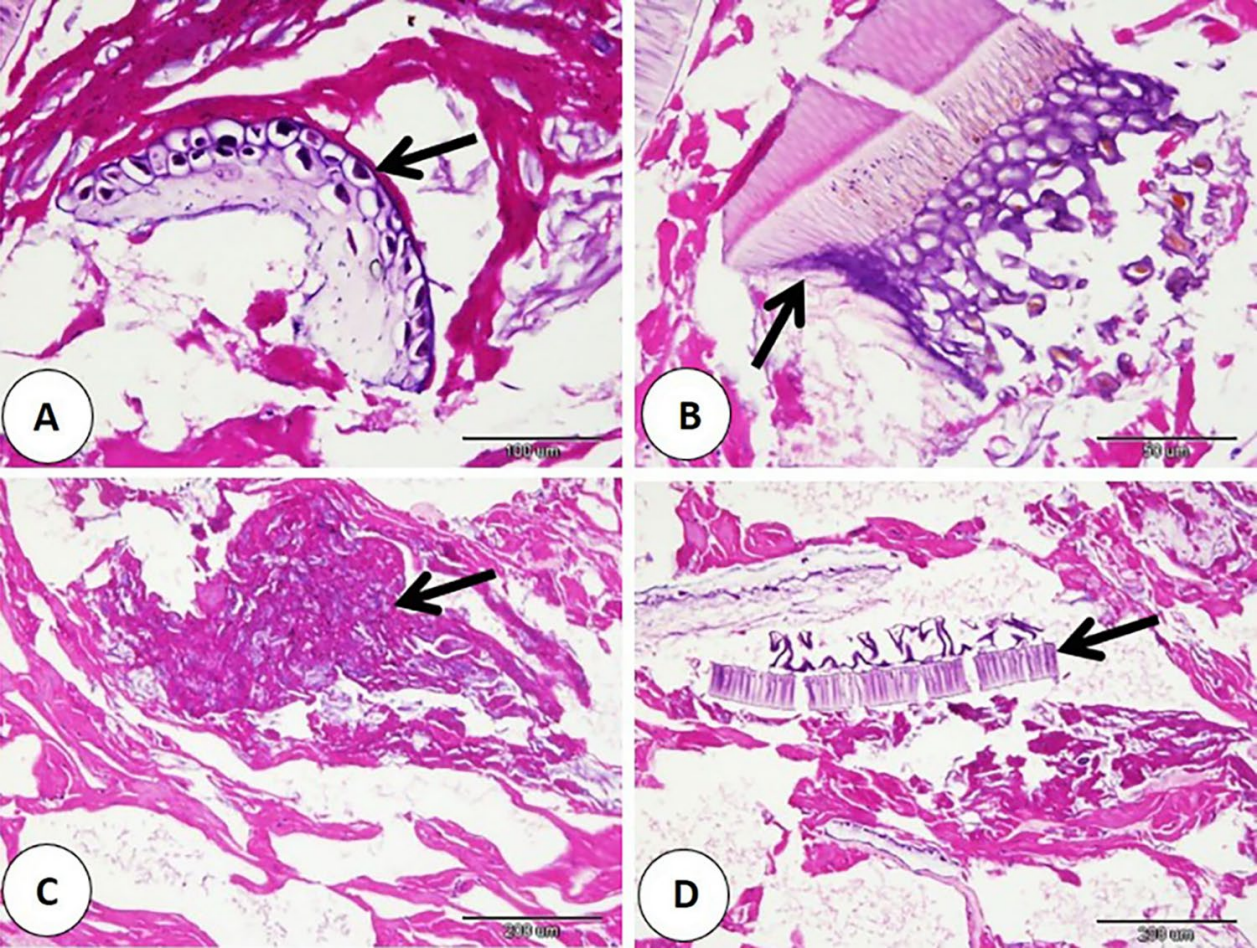
Samples of minced meat showed adulteration with different parts of plant tissues (arrows). H&E stain.

Our data indicated that various percentages of animal tissues as adipose tissue, collagen fibers, bone, cartilage, and smooth muscle fibers detect in different products. The highest percentage of animal tissues were observed in samples of kofta which significantly increase than those observed on other products. On the other hand, the lowest percentage of animal tissues were detected in samples of kofta and significantly decrease than those detected on other products (fig. 1A-B-C-D-E, 3,5,7B-C-D) and (Table 3). Examination of current samples detected various percentages of plant tissues within the sausage, burger, kofta, and minced meat samples. The highest percentage of plant tissues detected in samples of the burger which significantly increase than those observed in the samples of the kofta and minced meat but increase non-significantly than those observed in sausage. However, minced meat samples shoed the lowest percentage of plant tissues which decrease significantly than those detected on sausage and burger ( fig. 2,4,6,8) and (Table 3).

**Fig. 9:**
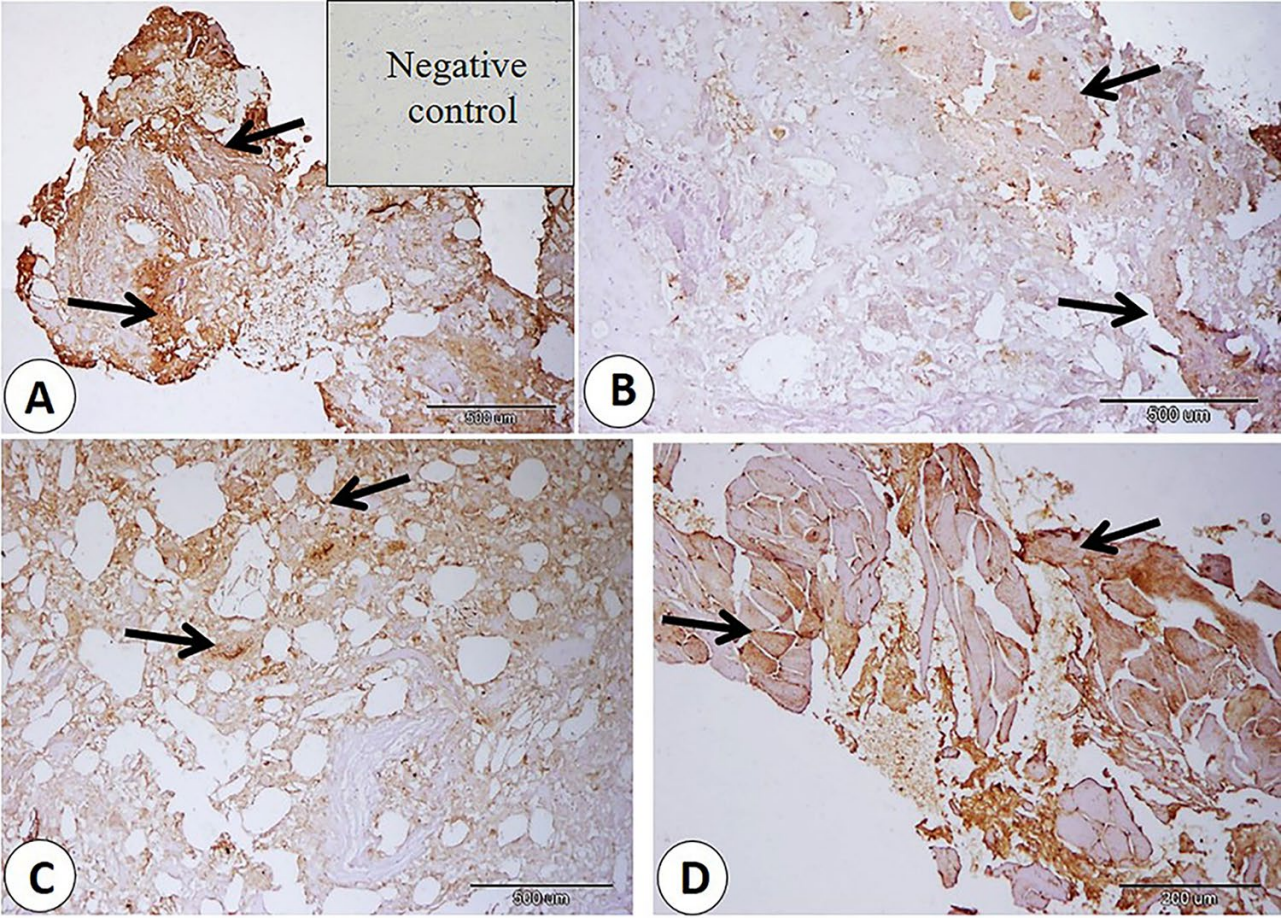
BCL2 expression (arrows). **A**: Bcl2 in sausage samples. **B**: Bcl2 in burger samples. **C**: Bcl2 in kofta samples. **D**: Bcl2 in minced meat samples. Note, the negative control is represented in the bordered area in A.

The current immunohistochemistry data showed different percentage intensity in Bcl2 expression among sausage, burger, kofta, and minced meat samples. The highest percentage intensity of Bcl2 expression demonstrated in sausage samples and the lowest percentage intensity of Bcl2 expression detect in burger samples. In addition, there were significance decrease in the percentage intensity of Bcl2 expression in burger samples than other products (fig. 9) and (Table 4)

**Table 3:** Percentage (%) of SKM, animal tissues, and plant tissues within sausage, burger, Kofta, and minced meat.

Kind of product	SKM Percentage (%)	Animal tissues Percentage (%)	Plant tissues Percentage (%)
Sausage	44.92 ± 2.97^b^	40.59 ± 2.80^b^	14.49 ± 3.27^a^
Burger	45.45 ± 0.91^b^	33.83 ± 1.01^b^	20.72 ±.14^a^
Kofta	7.43 ± 2.05^c^	85.39 ± 4.54^a^	7.18 ± 2.86^b^
Minced meat	82.10 ± 1.74^a^	15.21 ± 1.83^c^	2.69 ± 0.13^b^

**Table 4:** Percentage of Bcl2 intensity in sausage, burger, Kofta, and minced meat.

product	Percentage intensity of BCL2 (%)
Sausage	27.12 ± 0.42^a^
Burger	18.17 ± 2.15^b^
Kofta	25.23 ± 0.16^a^
Minced meat	26.60 ± 2.75^a^

Means within the same column with different superscripts differ significantly [*p* < 0.05].

The current study planned to use PCR to identify DNA fingerprint of animal species in different 46 meat products and the results revealed that 89.13% (41/46) were pure beef products and 10.87% (5/46) were contaminated with rodents. 100% of analyzed samples were negative for canine species.

**Fig. 10:**
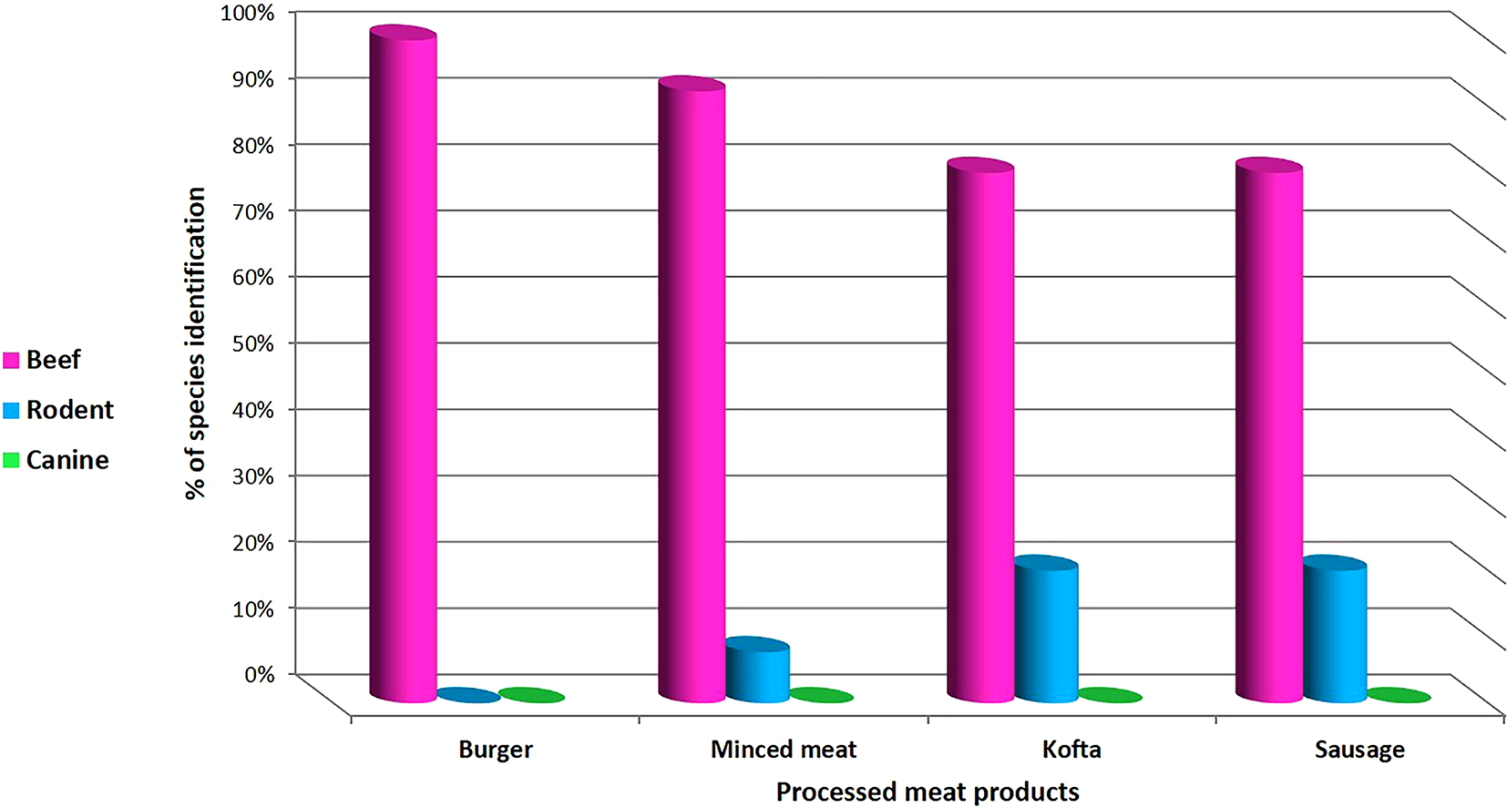
Percent of species identification in different meat products.

**Fig. 11:**
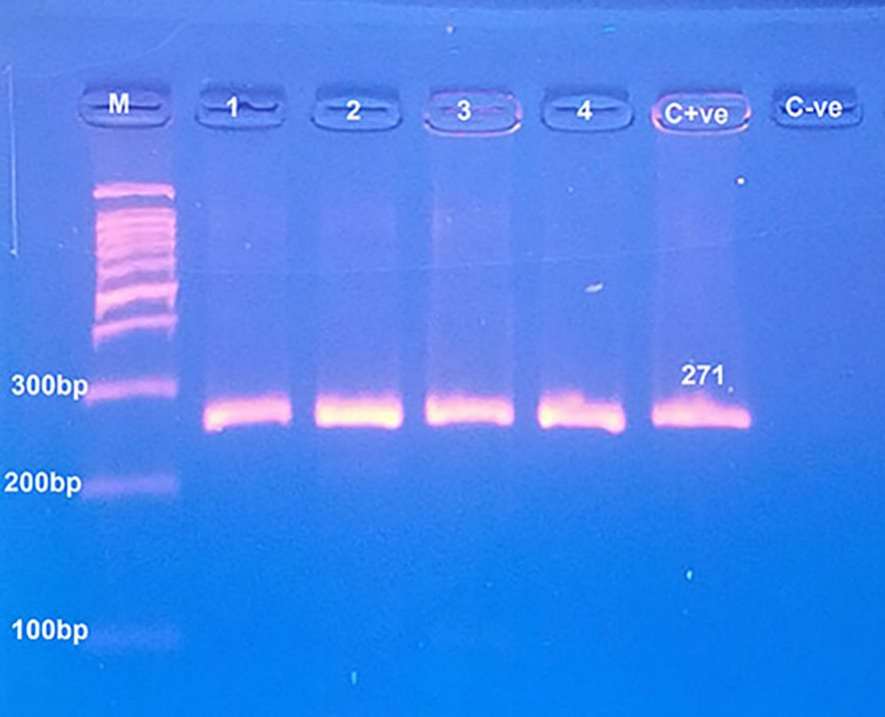
Agarose gel electrophoresis of PCR showed pure beef products samples (lines with No. 1, 2, 3 and 4) with control positive (C + ve) at 271 bp, control negative (C-ve) and line M: DNA ladder 100 bp.

**Fig. 12:**
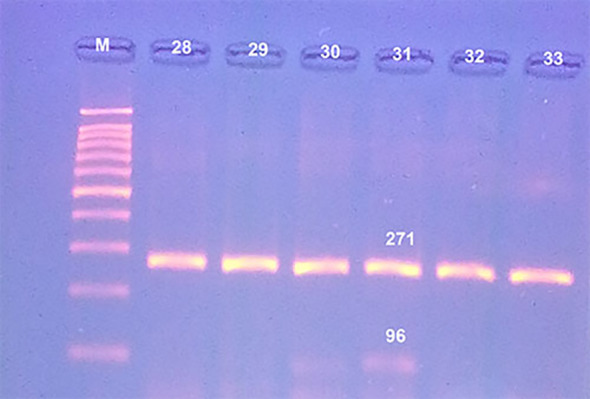
Agarose gel electrophoresis of PCR showed pure beef products samples (lines with No. 28, 29, 32 and 33) at 271 bp and rodents species contaminated beef products sample with line No. 31 at 96 bp where line M: DNA ladder 100 bp.

**Fig. 13:**
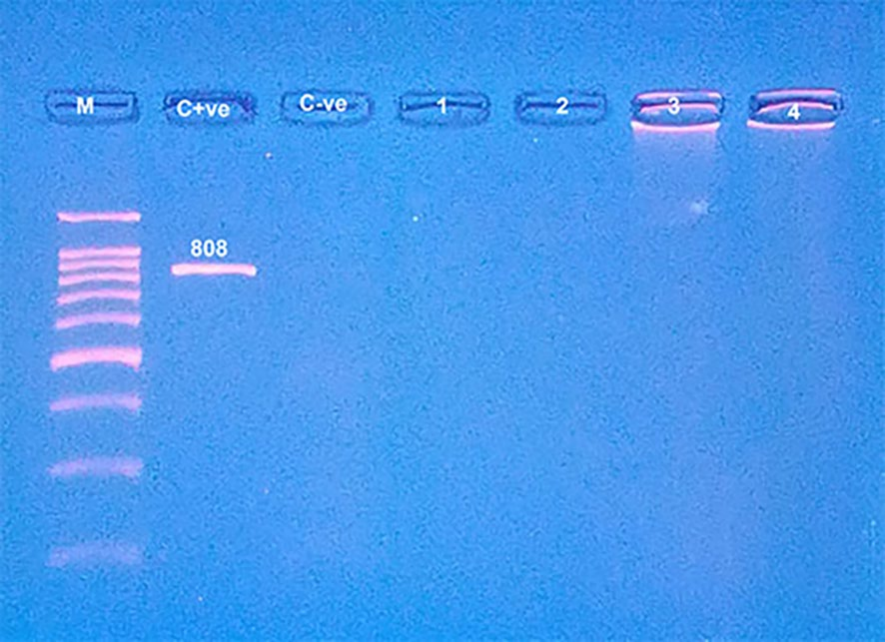
Agarose gel electrophoresis of PCR showed products free from canine tissues with line No. 1, 2, 3 and 4 with control negative (C-ve) and control positive (C + ve) at 808 bp, where line M: DNA ladder 100 bp.

**Fig. 14:**
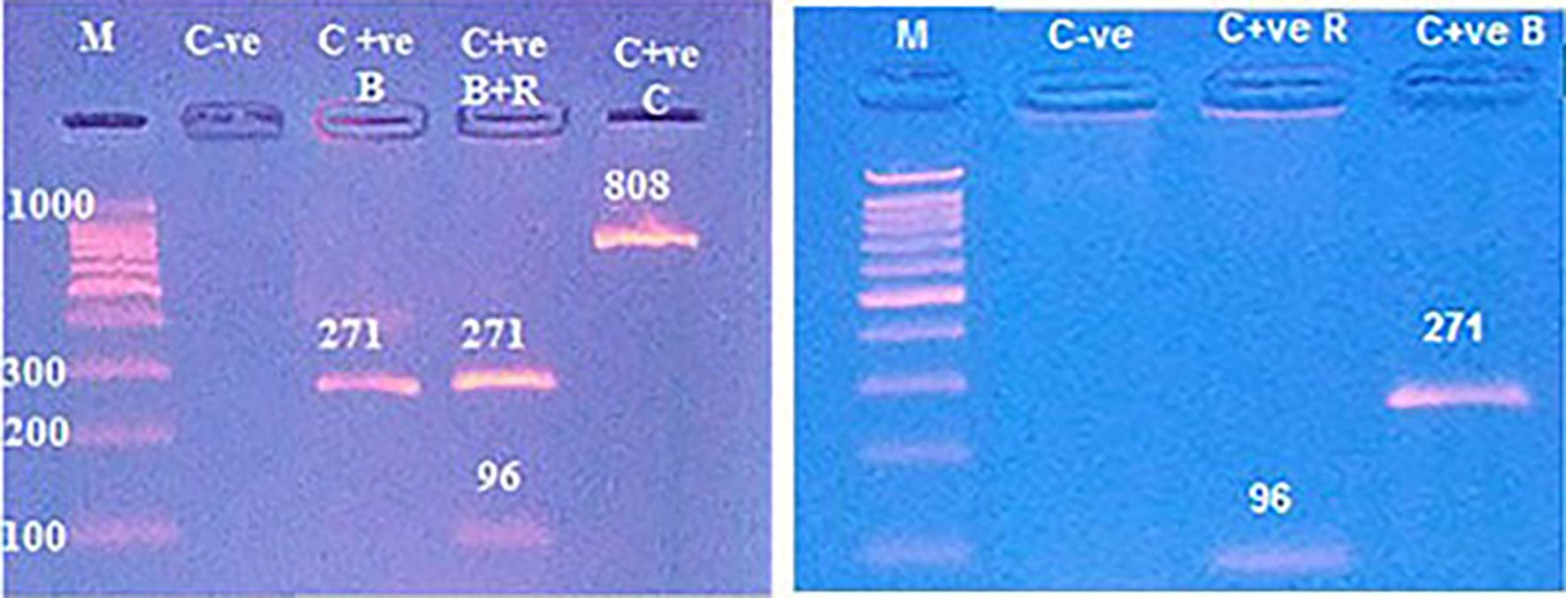
Agarose gel electrophoresis of PCR showed control negative (C-ve) and control positive (C + ve) of bovine (B), rodents (R) and canine (C) at 271, 96 and 808 bp, where line M: DNA ladder 100 bp.

Pure bovine samples included 100% (13/13), 92.31% (12/13), 80% (8/10) and 80% (8/10) of burger, minced meat, kofta and sausage, respectively (Table 5),and (fig. 10 and fig. 11). The five samples which were contaminated with rodents meat were as the following: 0% (0/13), 7.69% (1/13), 20% (2/10) and 20% (2/10) of burger, minced meat, kofta and sausage, respectively (Table 5) and (fig. 12). Also, the current results showed that 100% of the analyzed samples were negative for canine according to Table 5 and fig. 13. All the previous results were compared with the negative and positive control of bovine, rodents and canines species (fig. 14).

**Table 5:** Species identification in different meat products.

Species /Meat product	Beef Burger	Minced meat	Beef Kofta	Beef Sausage
	**No. (%)**	**No. (%)**	**No. (%)**	**No. (%)**
Pure bovine meat	13 (100%)	12 (92.31%)	8 (80%)	8 (80%)
Bovine and rodents meat	0 (0%)	1 (7.69%)	2 (20%)	2 (20%)
Bovine and canine meat	0 (0%)	0 (0%)	0 (0%)	0 (0%)
Total (46 samples)	13	13	10	10

## Discussion

The rapid pace of life around the world and the large number of working hours have made the use of meat products a reality, and this has been supported by the great progress witnessed in the meat products sector. At the same time, greedy people adulterate these products, seeking to make a quick profit without caring about the quality of the products, which directly reflects on human health. This fraud is based on the use of cheap plant or animal tissues in large and unauthorized quantities to reduce the percentage of meat in the products.

Adulteration of meat products in Egypt was a common problem highlighted by several researchers. Adulteration was detected between 8 and 60% in beef-based meat products with pigs, donkeys, camels, and chicken tissues^29^. Also, 25–50% of examined beef product samples were adulterated with pork flesh^39^. Moreover, soy protein is detected with percentage of 64–97% in different meat products collected from supermarkets in Egypt^40^.

Evaluation of meat products with histological techniques is used previously as an effective method for evaluating meat quality quantitatively and qualitatively and has helped detect unauthorized tissues in various meat products^29,41,42^. The cooking and heating do not restrict the histological detection of the meat constituent^14,43^. Our histological analysis of sausage, burger, kofta, and minced meat highlighted varying degrees of adulteration with animal and plant tissues. The highest percentage of skeletal muscle was detected in samples of minced meat, so it is the least product in adulteration. In contrast, the lowest skeletal muscle content was detected in the samples of the kofta, which means more adulteration with non-muscle tissues, as adipose tissue, collagen fibers, bone, cartilage, and smooth muscle fibers. So, several products analyzed histologically in this study did not follow the Egyptian Organization for Standardization and Quality Control (EOS), which specifies that beef-based products should be devoid of cartilage, bones, ligaments, tendons, stomach, and intestines^29^.

Regarding animal tissue composition, kofta contained the highest proportion of animal tissues as adipose tissue, collagen, cartilage, and smooth muscle fibers. On the other hand, minced meat showed the lowest content of animal tissues. Also, sausages and burger samples showed intermediate levels of animal tissues. The high collagen content in meat products is associated with increased toughness^44^. The presence of cartilage in meat is an indicator of lower quality and reduced nutritional value. Also, fragments of bone are not naturally present in meat products; their presence is an indication of a problem in meat or contaminated with fetal tissues^14,45^. Additionally, the quality of meat products tends to decline as fat content rises. Several studies using histological analysis and detect unauthorized collagen, adipose tissues, cartilage, bone, and parts of hollow organs in meat products in Egypt and all over the world^29,46^. Histological examination described that the presence of plant tissues was most distinct in burger and sausage samples, which contained significantly more plant material compared to kofta and minced meat. Minced meat exhibited the lowest proportion of plant tissue our result agrees with^29^ who described that the lowest amount of plant tissue detected on samples of minced meat when examined minced meat, kofta, sausage, burger, and luncheon. The higher percentage of plant tissues in other product rather than minced meat may be due to giving the texture, and flavor of this products.

The use of plant tissues in sausages and burgers appears to be a cost-saving strategy. The unauthorized addition of high levels of plant tissues can decrease both the quality and nutritional value of meat products^47^. According to the Egyptian Organization for Standardization and Quality Control (EOS) guidelines, the soy content in meat products should not exceed 10%, in line with national standards. While soy protein is recognized for its potential health benefits, such as lowering plasma cholesterol and reducing the risk of diabetes and cancer^48^. Some studies discussed the negative effects of excessive soy consumption, including organ toxicity and hormonal problems^49^. In meat products, any additive must be declared on the label of the ingredients^6^. Certain plant materials, such as onion, soy protein, breadcrumbs …etc., are commonly used in the production of meat products^50–52^. However, we acknowledge the importance of fully disclosing all ingredients, especially those present in high percentages.

In addition, the detection of sarcocysts in some samples raises concerns about standards, highlighting the need for strict quality control.

Our immunohistomorphometrical data demonstrated variations in Bcl2 expression across sausage, burger, kofta, and minced meat samples. According to our data Sausage samples showed the maximum Bcl2 expression, while burger samples showed the minimum. On the other hand, Kofta and minced meat exhibited intermediate levels of Bcl2 expression. The overall decrease in Bcl2 expression across products may be related to oxidative stress or damage induced by preservation and preparation methods^53,54^. Programmed cell death, or apoptosis, occurs because of tissue anoxia following death, which is linked to the cessation of blood flow. Apoptosis is regulated by both pro-apoptotic and anti-apoptotic members of the BCL2 protein family, which plays a key role in regulation of cell death (BCL2)^55,56^. These proteins are notable by their capability to form pores in the mitochondrial membrane and other intracellular membranes, an important mechanism in the regulation of mitochondrial integrity during apoptosis^57–59^.

PCR is also considered the most sensitive monitoring test to detect animal species substitution for religious and health importance^23^. Results of the current study showed that 100% of the analyzed samples were positive for beef and negative for canine but also displayed that 89.13% of analyzed meat samples were pure beef products and 10.87% were contaminated with rodents. The presence of rodent species in different analyzed beef products has two sources, the first source was from unhygienic measures and the presence of rodents in the equipment during manufacture or processing steps of the products^10^. The second source is linked to the adulteration of meat products with soybeans contaminated with rodent’s excretions. Soybean is used recently as a substitute in meat products thanks to its high protein level and cheap price^60^.

Meat products adulteration were detected in previous studies in different countries, including Egypt. The current study results agreed with previous studies that revealed that 100% of the examined meat products were positive for beef and free from canine tissue^61^. Partially agreed with the study done in Cairo governorate, Egypt^27^, although the examined commercial meat products were labeled as 100% beef, 16.4% of meat products samples were negative for bovine species, and 9.2% of the analyzed products were adulterated with rodents, especially in kofta and sausage, and disagreed with the study in Kalubia governorate^26^, which detected that canine meat was detected in 33.3 and 66.7% of the examined kofta and sausage samples, respectively.

## Conclusion

This study described significant adulteration in different commercial meat products through histological, immunohistochemical and PCR techniques. Samples of kofta contained a higher percentage of animal tissues rather than skeletal muscles. Burger samples were the most abundant in plant tissues. A key novelty in the present work was the use of Bcl2 immunohistochemistry, which confirmed differences in tissue integrity. The highest Bcl2 expression detected in sausage, suggesting superior quality of the muscle, while the lowest Bcl2 expression detected in burger samples, suggesting degradation. These data highlight the novel use of Bcl2 immunohistochemistry as an effective, reliable method assessing meat product quality, offering new tools for food safety and quality control. Also, the current study evaluated the potential adulteration and/or contamination of some meat products with rodents and canine species and ensured its beef origin with PCR.

## Electronic supplementary material

Below is the link to the electronic supplementary material.

Supplementary Material 1

## Data Availability

The data generated during and/or analyzed during the current study are available from the corresponding author on reasonable request.
