# Supplementary material for: Histological, immunohistochemical assessment and DNA fingerprint species identification of some meat products in Egypt
Source: Sci Rep. 2025 Apr 29;15:14978. doi: 10.1038/s41598-025-97633-9 (PMC12041293; doi:10.1038/s41598-025-97633-9)
Supplement: Supplementary file 1 — Supplementary Material 1 [file 41598_2025_97633_MOESM1_ESM.docx]

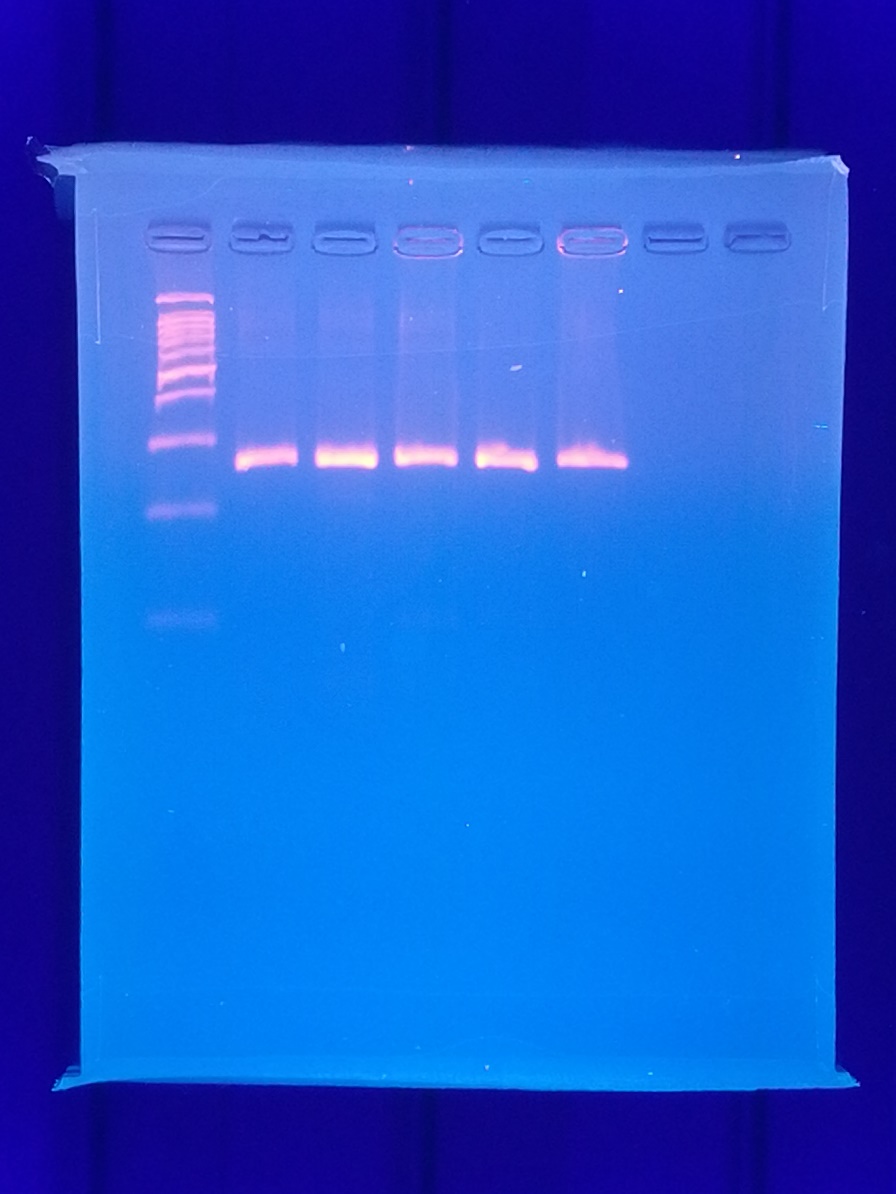


Figure S1. Agarose gel electrophoresis of PCR showed pure beef products samples with control positive and negative.


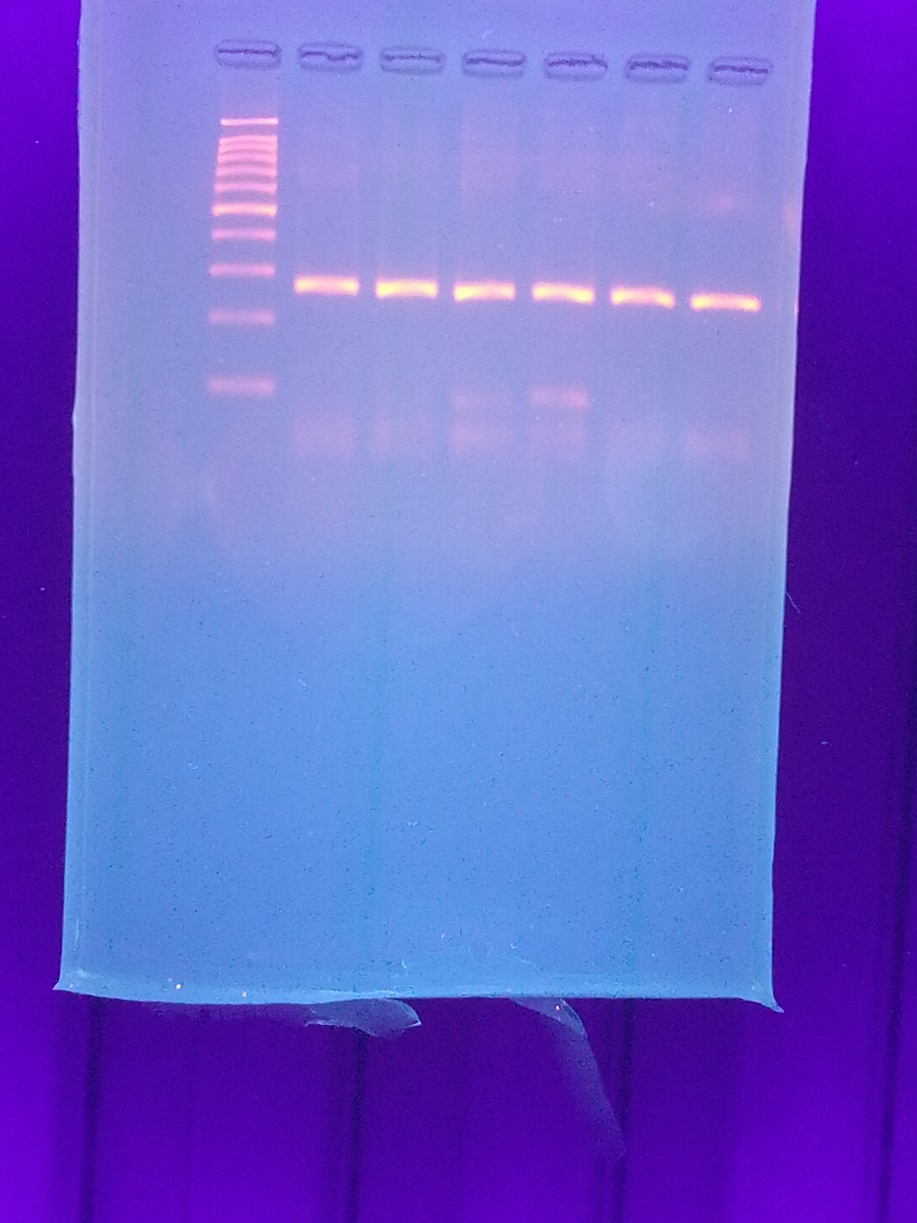


Figure S2. Agarose gel electrophoresis of PCR showed pure beef products samples and rodents species contaminated beef products.


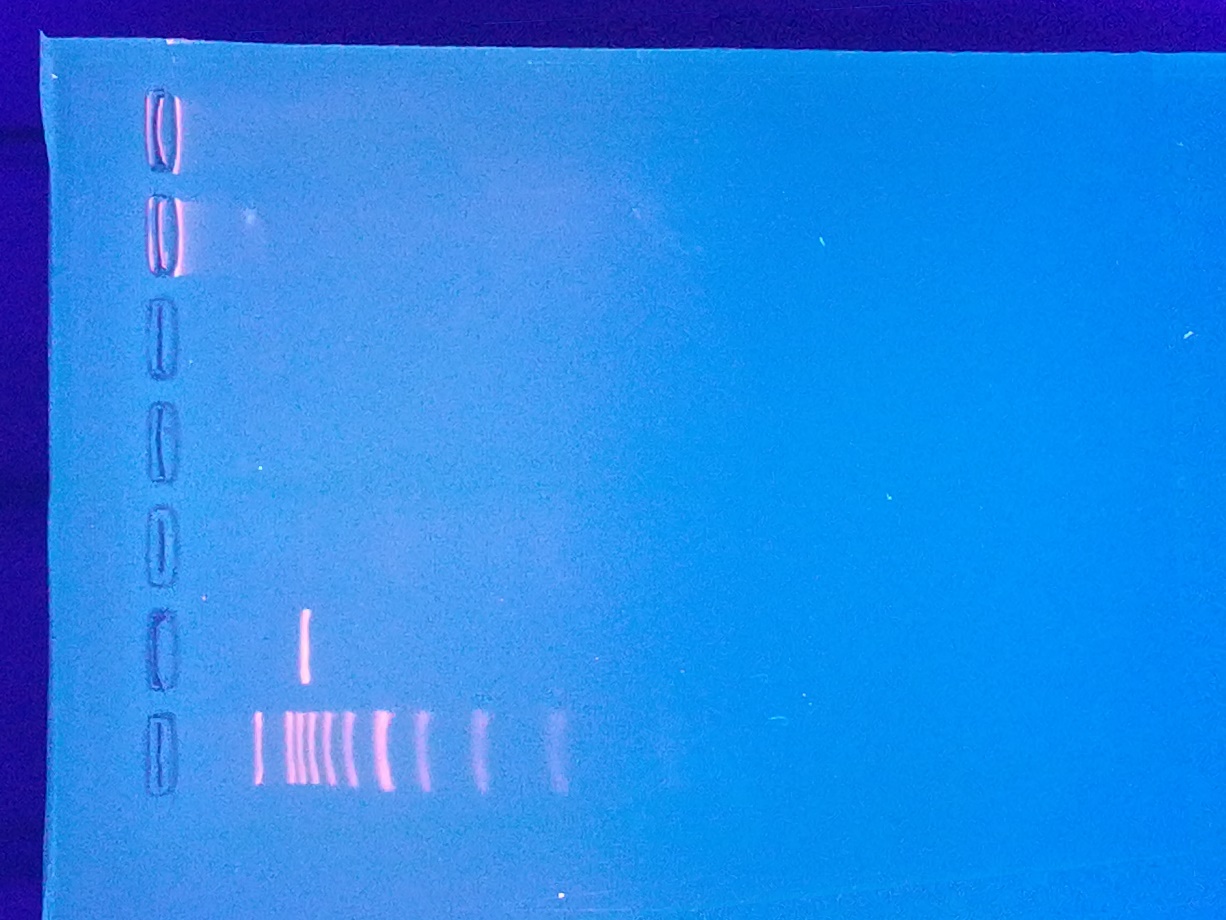


Figure S3. Agarose gel electrophoresis of PCR showed products free from canine tissues with its control positive and negative.


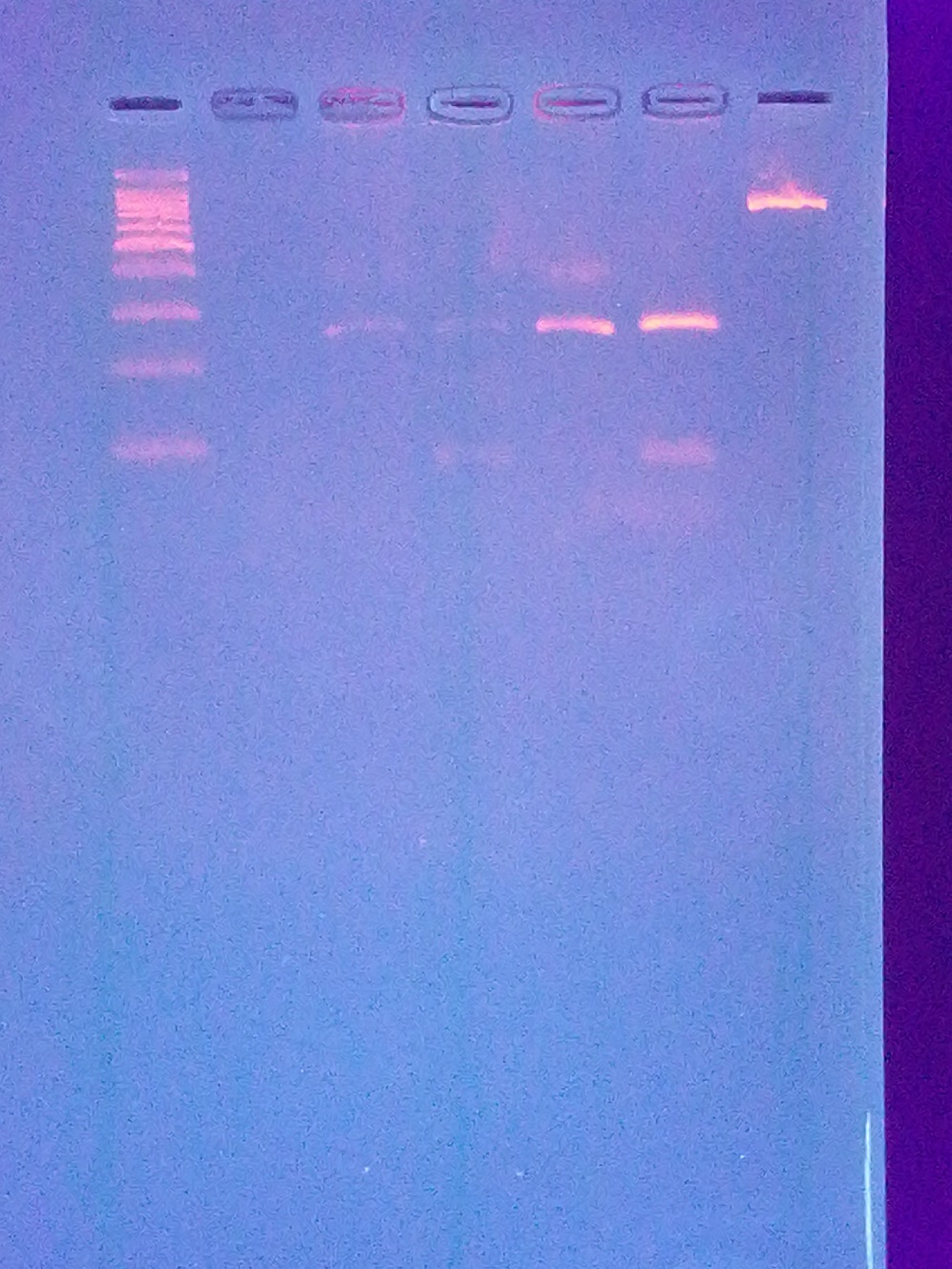


Figure S4. Agarose gel electrophoresis of PCR showed control negative and control positive of bovine , rodents and canine


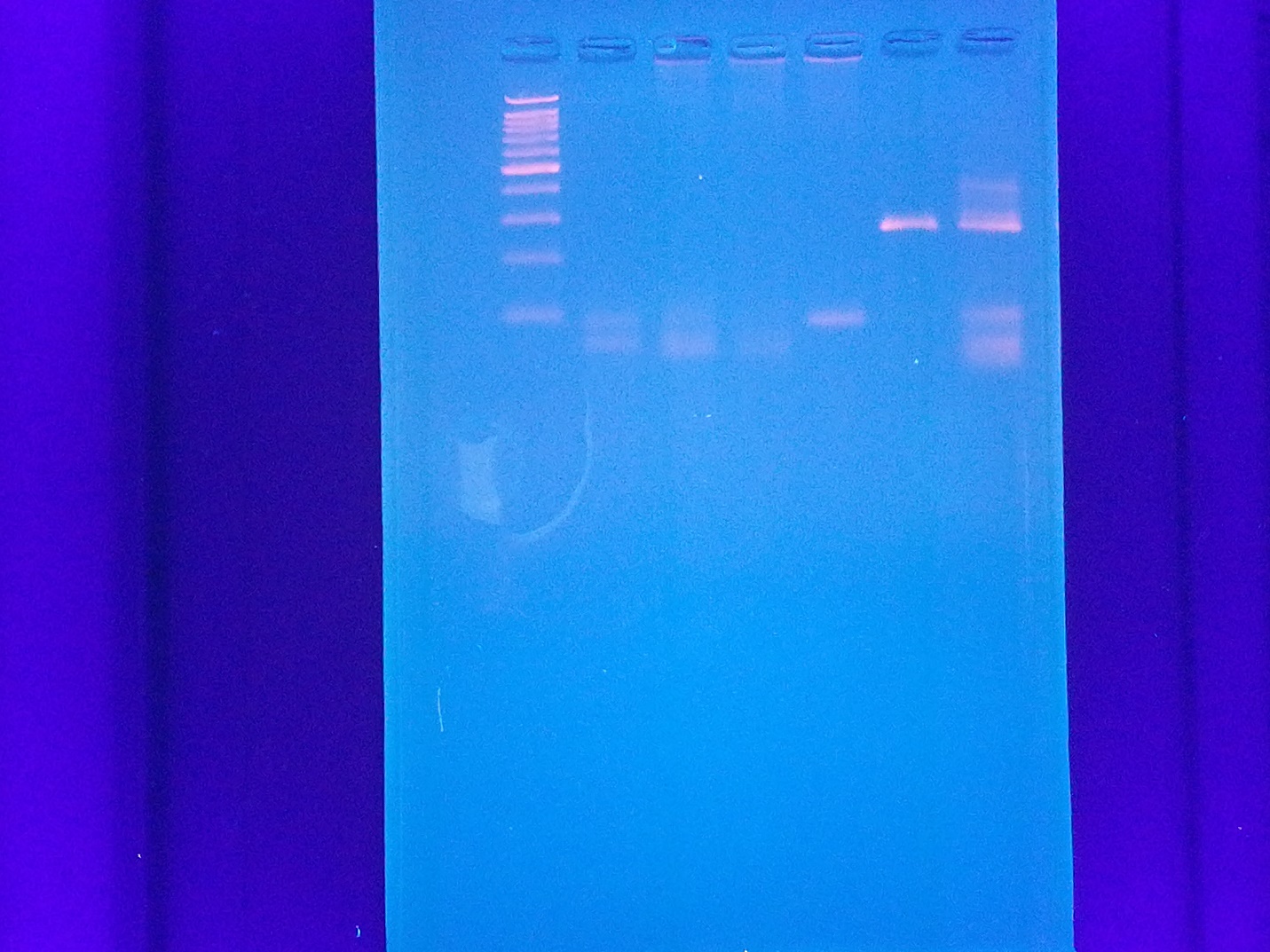


Figure S5. Agarose gel electrophoresis of PCR showed control negative and control positive of bovine and rodents
